# Treatment and Survival Outcomes Associated With Platinum Plus Low-Dose, Long-term Fluorouracil for Metastatic Nasopharyngeal Carcinoma

**DOI:** 10.1001/jamanetworkopen.2021.38444

**Published:** 2021-12-13

**Authors:** Shuo-Han Zheng, Song-Ran Liu, Hai-Bo Wang, Ying-Hong Wei, He Li, Guan-Nan Wang, Zi-Lu Huang, Shi-Rong Ding, Chen Chen, Ya-Lan Tao, Xiao-Hui Li, Christophe Glorieux, Peng Huang, Yang-Feng Wu, Yun-Fei Xia

**Affiliations:** 1 State Key Laboratory of Oncology in South China, Collaborative Innovation Center for Cancer Medicine, Guangdong Key Laboratory of Nasopharyngeal Carcinoma Diagnosis and Therapy, Sun Yat-sen University Cancer Center, Guangzhou, China; 2 Department of Radiation Oncology, Sun Yat-sen University Cancer Center, Guangzhou, China; 3 Department of Pathology, Sun Yat-sen University Cancer Center, Guangzhou, China; 4 Peking University Clinical Research Institute, Peking University First Hospital, Beijing, China; 5 Department of Head and Neck Surgery, Sun Yat-sen University Cancer Center, Guangzhou, China; 6 Department of Hematology and Endocrinology, The PLA 74th Group Army Hospital, Guangzhou, China; 7 Center for Cancer Metabolism and Intervention Research, Sun Yat-sen University Cancer Center, Guangzhou, China

## Abstract

**Importance:**

The treatment of metastatic nasopharyngeal carcinoma (mNPC) is a major challenge because of drug resistance and the toxic effects of chemotherapy.

**Objective:**

To evaluate the survival and toxicity outcomes and safety associated with the use of a modified low-dose fluorouracil protocol compared with standard regimens recommended in current guidelines for treatment of mNPC.

**Design, Setting, and Participants:**

This retrospective cohort study was based on data retrieved from electronic medical records from Sun Yat-sen University Cancer Center in China for 1397 patients with mNPC diagnosed from January 1, 2006, to December 31, 2017. Data analyses were conducted from October 1, 2020, to May 1, 2021.

**Exposures:**

Patients received chemotherapy, including platinum plus low-dose, long-term fluorouracil (PFLL); cisplatin plus standard dose, short-term fluorouracil (PFSS); cisplatin plus gemcitabine (GP); cisplatin plus taxane (TP); and cisplatin plus taxane plus fluorouracil (TPF).

**Main Outcomes and Measures:**

The main outcomes included overall survival (OS); subsequent-line, treatment-free survival (sTFS), defined as the period from metastasis to the date requiring subsequent-line treatment or death; and the survival to toxicity ratio (STR), defined as person-year rate of OS divided by person-year rate of severe hematologic toxic effects. Cox regression models were used to compare the outcomes of patients receiving PFLL vs other regimens, adjusting for baseline characteristics.

**Results:**

Of 1397 patients with mNPC included in this study (1152 men; median age, 46 years [range, 18-70 years]) 134 received PFLL, 203 received GP, 330 received PFSS, 366 received TP, and 364 received TPF. A total of 764 patients died (75 in treatment group PFLL; 107 in group GP; 204 in group PFSS; 207 in group TP; and 171 in group TPF), and 979 patients had subsequent-line treatment or died, whichever occurred first (PFLL, 77; GP, 144; PFSS, 262; TP, 269; and TPF, 227). The median follow-up was 46.9 months (IQR, 25.4-82.4 months), and the 5-year OS rate among patients who received PFLL was 25.4% (95% CI, 16.7%-38.8%), which was not significantly different from that among patients who did not receive PFLL (30.2%; 95% CI, 27.1%-33.5%; *P* = .13) or who received GP (25.1%; 95% CI, 18.1%-35.0%; *P* = .81), PFSS (23.6%; 95% CI, 18.5%-30.0%; *P* = .80), or TP (28.1%; 95% CI, 22.8%-34.7%; *P* = .99) but was lower than that for patients who received TPF (40.4%; 95% CI, 34.7%-47.1%; *P* = .001). The 5-year sTFS among patients who received PFLL (24.1%; 95% CI, 15.4%-37.6%) was significantly higher than that among patients who did not receive PFLL (18.5%; 95% CI, 16.1%-21.3%; *P* = .005) or who received GP (14.3%; 95% CI, 9.1%-22.5%; *P* = .001) but similar to that for patients who received TPF (28.0%; 95% CI, 23.0%-34.0%; *P* = .74). The STR of PFLL was 0.81, substantially better than that of GP (0.41) and TPF (0.65).

**Conclusions and Relevance:**

The results of this cohort study suggest that, compared with the use of standard treatment regimens, administration of PFLL was associated with similar OS but prolonged sTFS. PFLL also had better STR than other regimens, which could indicate less severe toxic effects. Thus, PFLL may be an option for first-line treatment of mNPC.

## Key Points

**Question:**

What are the survival and toxicity outcomes associated with platinum plus low-dose, long-term fluorouracil compared with other chemotherapy regimens?

**Findings:**

This cohort study of 1397 consecutive patients from 2006 to 2017 with metastatic nasopharyngeal carcinoma found that administration of platinum plus infusion of low-dose, long-term fluorouracil was associated with similar overall survival but better subsequent-line, treatment-free survival than other conventional chemotherapy regimens.

**Meaning:**

Findings from this study suggest that platinum plus low-dose, long-term fluorouracil may be an optional treatment regimen to control disease progression for patients with metastatic nasopharyngeal carcinoma.

## Teaser

This cohort study uses real-world evidence to assess the survival outcomes and hematologic toxic effects associated with the use of a low-dose, long-term infusion protocol for administration of fluorouracil vs standard regimens recommended in current guidelines for treatment of patients with metastatic nasopharyngeal carcinoma.

## Introduction

Nasopharyngeal carcinoma (NPC) is a malignant tumor of the nasopharyngeal epithelium with high sensitivity to ionizing radiation and chemotherapy.^1^ According to its clinical features, the type of NPC is classified as ascending, descending, or mixed,^2^ and NPC is categorized as not metastatic prone (70%-80%) or metastatic prone (20%-30%).^3^ The primary treatment of NPC is radiotherapy with or without chemotherapy, which can achieve excellent local control but leaves distant metastasis as a major challenge and a main cause of failure.^4,5,6,7,8,9,10^

The outcome of patients with metastatic NPC (mNPC) is poor, with a median overall survival (OS) of approximately 20 months.^11^ Chemotherapy is the foundation of mNPC treatment. Platinum-based chemotherapy and single-agent chemotherapy are recommended by the National Comprehensive Cancer Network guidelines, and the recommended regimens are updated periodically. Current standard regimens include the use of cisplatin plus fluorouracil (PF), cisplatin plus gemcitabine (GP), cisplatin plus taxane (TP), and cisplatin plus taxane plus fluorouracil (TPF). In the past, PF was widely used as first-line chemotherapy because of its good antitumor activity and tolerable toxicity; however, a new option was recommended after a multicenter phase 3 randomized clinical trial reported the results in 2016. In that trial, GP significantly prolonged OS, with 29.1 months vs PF with 20.9 months.^12^ However, the incidence of severe treatment-related adverse events was significantly higher for the use of GP than for PF. Triple combination regimens, such as TPF, showed excellent short-term efficacy, but were also associated with severe toxic effects, even death.^13,14^ Thus, the TP regimen was used to reduce toxic effects, and it showed excellent long-term efficacy, clearly better than GP.^13,15^

However, the clinical responses to these chemotherapy regimens are not always satisfactory. Poor outcome was observed in a subset of patients and may be due in part to chemoresistance and treatment-related toxic effects, which may debilitate patients and impede their adherence to the treatment schedule. Some studies indicated that low-dose chemotherapy may reduce toxic effects while maintaining or even improving efficacy, compared with the conventional dose of chemotherapy used for treatment of metastatic carcinoma.^16,17,18,19^ For recurrent or metastatic NPC, a phase 2 trial of fluorouracil infusion at a dose of 300 mg/m^2^ for 6 consecutive weeks found a median survival of 10 months and mild toxic effects.^18^ Fluorouracil is a cell cycle–dependent drug whose cytotoxicity is proportional to the cell proliferation rate and to the drug exposure time.^19,20^ A continuous intravenous infusion of fluorouracil at a constant rate may maintain a stable blood drug concentration, and a long-term infusion may overcome the short half-life of fluorouracil (approximately 20 minutes), which may limit the exposure of cycling cells to the drug.^20,21,22^ Therefore, we postulated that the use of a long-term infusion of low-dose fluorouracil would be associated with improved survival outcomes and low toxicity to thus be associated with enhanced overall therapeutic benefit. In 2006, we started a research project supported by the National High Technology Research and Development Program in China in which patients with mNPC were treated with a modified PF regimen. The regimen comprised administration of platinum plus a continuous intravenous infusion of low-dose, long-term fluorouracil (PFLL, also known as “Lao Huo Bao Tang Fa” in Chinese). Since then, this research project has accumulated a wealth of data with respect to survival, toxic effects, and other clinical parameters.

Although randomized clinical trials represent the criterion standard for therapeutic evaluation, there is growing interest in real-world evidence to overcome certain limitations associated with clinical trials.^23^ In the present cohort study, we compared data generated from the PFLL project with other relevant clinical data from the same institution (Sun Yat-sen University Cancer Center [SYSUCC]), aiming to find a favorable therapeutic option among the common platinum-based regimens for treatment of mNPC.

## Methods

### Study Population

We identified 1930 patients with mNPC consecutively during a 12-year period from January 1, 2006, to December 31, 2017, at SYSUCC. The inclusion and exclusion criteria are given in eFigure 1 in the Supplement. All patients were either enrolled in or outside clinical trials. The relevant clinical trials are summarized in eTable 1 in the Supplement. This study is registered at ClinicalTrials.gov.^24^ Data are reported in a manner consistent with the Strengthening the Reporting of Observational Studies in Epidemiology (STROBE) reporting guidelines. This retrospective cohort study was approved by the institutional review board of SYSUCC, which waived the requirement for obtaining informed consent because the study was considered a secondary analysis of existing data of deidentified patients. No one received compensation or was offered any incentive for participating in this study.

### Treatment Groups

The first-line chemotherapy regimens in this retrospective study included PFLL, GP, cisplatin plus fluorouracil at a standard dose for a short term (PFSS), TP, and TPF (eTable 2 in the Supplement). Patients were grouped based on the regimen received (even when the patient died within the first cycle of a given chemotherapy; for example, patients in the PFLL group whose deaths occurred within the first 30 days). The treatment was delivered according to clinical protocols, clinicians’ suggestions, or the patient’s condition and willingness. Other types of therapy or supportive treatment that were permitted included local treatment (eg, radiotherapy, surgery, and radiofrequency ablation), molecular-targeted therapy, and immune checkpoint therapy.

### Data Collection and Follow-up

Demographic, clinical, and follow-up data were collected with database templates from electronic medical records and included patient gender, pathology results, date of the primary diagnosis, date of metastasis, age, baseline body mass index (BMI; calculated as weight in kilograms divided by height in meters squared), T and N stage according to the seventh edition of the American Joint Committee on Cancer and the International Union for Cancer Control system when metastasis was diagnosed, treatment failure number and reason before metastasis; and sites and number of metastases. Information on first-line therapy and subsequent-line therapies was obtained, including the start date and chemotherapy regimen, start date and sites of radiotherapy, the use of molecular-targeted therapy or immunotherapy, hematologic toxic effects evaluated with the National Cancer Institute Common Terminology Criteria for Adverse Events, version 4.0, and tumor responses assessed with Response Evaluation Criteria in Solid Tumors, version 1.1. Survival status was ascertained from follow-up medical clinic records of the hospital information system or by contacting the patients or their families by telephone or by data obtained from the official birth and death registration databases in China. Follow-up started at the diagnosis of metastasis, and the last follow-up date was December 31, 2018.

### Outcome Measures

The clinical end points analyzed included the following: (1) OS, defined as the time from metastasis to death from any cause or censored at the last visit or the final follow-up date; (2) subsequent-line therapy, treatment-free survival (sTFS), defined as the period from metastasis to the date requiring subsequent-line treatment or death from any cause or censored at the last visit or the final follow-up date, whichever came first; (3) cumulative incidence rate of severe hematologic toxic effects, including grade 3 or higher leukopenia, neutropenia, or thrombocytopenia, and the median interval from initiation of treatment to the first occurrence, measured using Kaplan-Meier estimates; (4) survival to toxicity ratio (STR), calculated by dividing the person-year rate of OS by the person-year rate of severe hematologic toxic effects (person-year rate was calculated by dividing the number of individuals who developed the outcome of interest during the observation period by the total amount of observation time in years of all patients); and (5) radiotherapy rate, the cumulative incidence rate of radiotherapy during the period of the first line of treatment.

### Statistical Analysis

Patients lost to follow-up were considered censored data, and all patients were included in survival analyses. The comparison between PFLL and non-PFLL groups was performed first and then pairwise comparisons between PFLL and any other regimen were conducted. The differences between categorical variables were compared using the χ^2^ test. Differences in STRs were compared using the numerical values. The median follow-up time was measured with the reverse Kaplan-Meier method. Survival curves were evaluated using the Kaplan-Meier method with the log-rank test. Univariable and multivariable Cox proportional-hazards models were used to estimate hazard ratios (HRs) and 95% CIs, adjusting for baseline variables. Subgroup analyses of survival by baseline factors were conducted with multivariable Cox proportional hazards models. Multiple imputation was used for missing values of baseline BMI, and the sensitivity analysis was then performed with a Cox regression model. However, patients with missing data for complete blood count or radiotherapy during treatment were excluded in the related analyses. Because pairwise comparisons between PFLL and any other regimen involved 4 comparisons, the definition of statistical significance was adjusted using the Bonferroni correction and defined as a 2-tailed α risk of .0125 or less to reduce the risk of making a type I error. All other analyses maintained the standard definition of statistical significance as a 2-tailed α risk of .05 or less. Statistical analyses were conducted from October 1, 2020, to May 1, 2021, with SPSS, version 24.0 (SPSS Inc), and R, version 4.0.2 (R Foundation for Statistical Computing).

## Results

This cohort study included 1397 patients, 1152 men (82.5%) and 245 women (17.5%), with a median age of 46 years (range, 18-70 years). The baseline characteristics of the patients in the various chemotherapy groups are given in Table 1. There were 134 patients (9.6%) treated with PFLL, 203 patients (14.5%) treated with GP, 330 patients (23.6%) treated with PFSS, 366 patients (26.2%) treated with TP, and 364 patients (26.1%) treated with TPF.

**Table 1.** Baseline Characteristics of Patients by Chemotherapy Received

Variable	Total No.	Patients, No. (%)					*P* values
		PFLL	GP	PFSS	TP	TPF	
All patients	1397	134 (100)	203 (100)	330 (100)	366 (100)	364 (100)	NA
Age, y							
<46	723	74 (55.2)	92 (45.3)	162 (49.1)	187 (51.1)	208 (57.1)	.06
≥46	674	60 (44.8)	111 (54.7)	168 (50.9)	179 (48.9)	156 (42.9)	
Sex							
Male	1152	113 (84.3)	169 (83.3)	286 (86.7)	288 (78.7)	296 (81.3)	.08
Female	245	21 (15.7)	34 (16.7)	44 (13.3)	78 (21.3)	68 (18.7)	
Year of metastasis							
2006-2011	495	6 (4.5)	31 (15.3)	148 (44.8)	144 (39.3)	166 (45.6)	<.001
2012-2017	902	128 (95.5)	172 (84.7)	182 (55.2)	222 (60.7)	198 (54.4)	
Pathology							
Keratinized	22	15 (11.2)	1 (0.5)	1 (0.3)	4 (1.1)	1 (0.3)	<.001
Nonkeratinizing	1375	119 (88.8)	202 (99.5)	329 (99.7)	362 (98.9)	363 (99.7)	
No. of treatment failures before metastasis							
0	587	52 (38.8)	39 (19.2)	167 (50.6)	130 (35.5)	199 (54.7)	<.001
1	748	69 (51.5)	148 (72.9)	160 (48.5)	219 (59.8)	152 (41.8)	
≥2	62	13 (9.7)	16 (7.9)	3 (0.9)	17 (4.6)	13 (3.6)	
T stage							
T0	701	68 (50.7)	136 (67.0)	146 (44.2)	213 (58.2)	138 (37.9)	<.001
T1	29	2 (1.5)	1 (0.5)	3 (0.9)	11 (3.0)	12 (3.3)	
T2	87	5 (3.7)	8 (3.9)	32 (9.7)	23 (6.3)	19 (5.2)	
T3	340	34 (25.4)	33 (16.3)	88 (26.7)	61 (16.7)	124 (34.1)	
T4	240	25 (18.7)	25 (12.3)	61 (18.5)	58 (15.8)	71 (19.5)	
N stage							
N0	647	49 (36.6)	119 (58.6)	146 (44.2)	195 (53.3)	138 (37.9)	<.001
N1	161	20 (14.9)	22 (10.8)	31 (9.4)	42 (11.5)	46 (12.6)	
N2	256	13 (9.7)	23 (11.3)	69 (20.9)	59 (16.1)	92 (25.3)	
N3	333	52 (38.8)	39 (19.2)	84 (25.5)	70 (19.1)	88 (24.2)	
Bone metastasis							
No	605	46 (34.3)	100 (49.3)	134 (40.6)	165 (45.1)	160 (44.0)	.07
Yes	792	88 (65.7)	103 (50.7)	196 (59.4)	201 (54.9)	204 (56.0)	
Liver metastasis							
No	929	95 (70.9)	116 (57.1)	224 (67.9)	241 (65.8)	253 (69.5)	.03
Yes	468	39 (29.1)	87 (42.9)	106 (32.1)	125 (34.2)	111 (30.5)	
Lung metastasis							
No	936	103 (76.9)	122 (60.1)	227 (68.8)	247 (67.5)	237 (65.1)	.02
Yes	461	31 (23.1)	81 (39.9)	103 (31.2)	119 (32.5)	127 (34.9)	
Distant lymph node metastasis							
No	968	85 (63.4)	114 (56.2)	237 (71.8)	257 (70.2)	275 (75.5)	<.001
Single region	339	36 (26.9)	71 (35.0)	74 (22.4)	85 (23.2)	73 (20.1)	
Multiple regions	90	13 (9.7)	18 (8.9)	19 (5.8)	24 (6.6)	16 (4.4)	
No. of metastatic sites							
Solitary	228	32 (23.9)	21 (10.3)	55 (16.7)	52 (14.2)	68 (18.7)	.009
Multiple	1169	102 (76.1)	182 (89.7)	275 (83.3)	314 (85.8)	296 (81.3)	
Baseline BMI							
Unknown	230	31 (23.1)	27 (13.3)	31 (9.4)	77 (21.0)	64 (17.6)	.61^a^
<23	785	70 (52.2)	114 (56.2)	202 (61.2)	204 (55.7)	195 (53.6)	
≥23	382	33 (24.6)	62 (30.5)	97 (29.4)	85 (23.2)	105 (28.8)	

Abbreviations: BMI, body mass index (calculated as weight in kilograms divided by height in meters squared); GP, cisplatin plus gemcitabine; NA, not applicable; PFLL, platinum plus continuous intravenous infusion of low-dose, long-term fluorouracil; PFSS, cisplatin plus fluorouracil with a short term and standard dose; TP, cisplatin plus taxane; TPF, cisplatin plus taxane plus fluorouracil.

^a^ Calculated for patients with records of baseline BMI.

### Five-Year Survival

The median follow-up for the whole cohort was 46.9 months (IQR, 25.4-82.4 months). A total of 219 patients (15.7%) were lost to follow-up before 5 years or the last follow-up date (14 patients treated with PFLL; 33 patients treated with GP; 51 patients treated with PFSS; 55 patients treated with TP; and 66 patients treated with TPF). In total, 764 patients (54.7%) died (75 patients treated with PFLL; 107 patients treated with GP; 204 patients treated with PFSS; 207 patients treated with TP; and 171 patients treated with TPF), and 979 patients (70.1%) had subsequent-line treatment or died, whichever occurred first (PFLL group, 77 patients; GP group, 144 patients; PFSS group, 262 patients; TP group, 269 patients; and TPF group, 227 patients).

**Figure 1.**
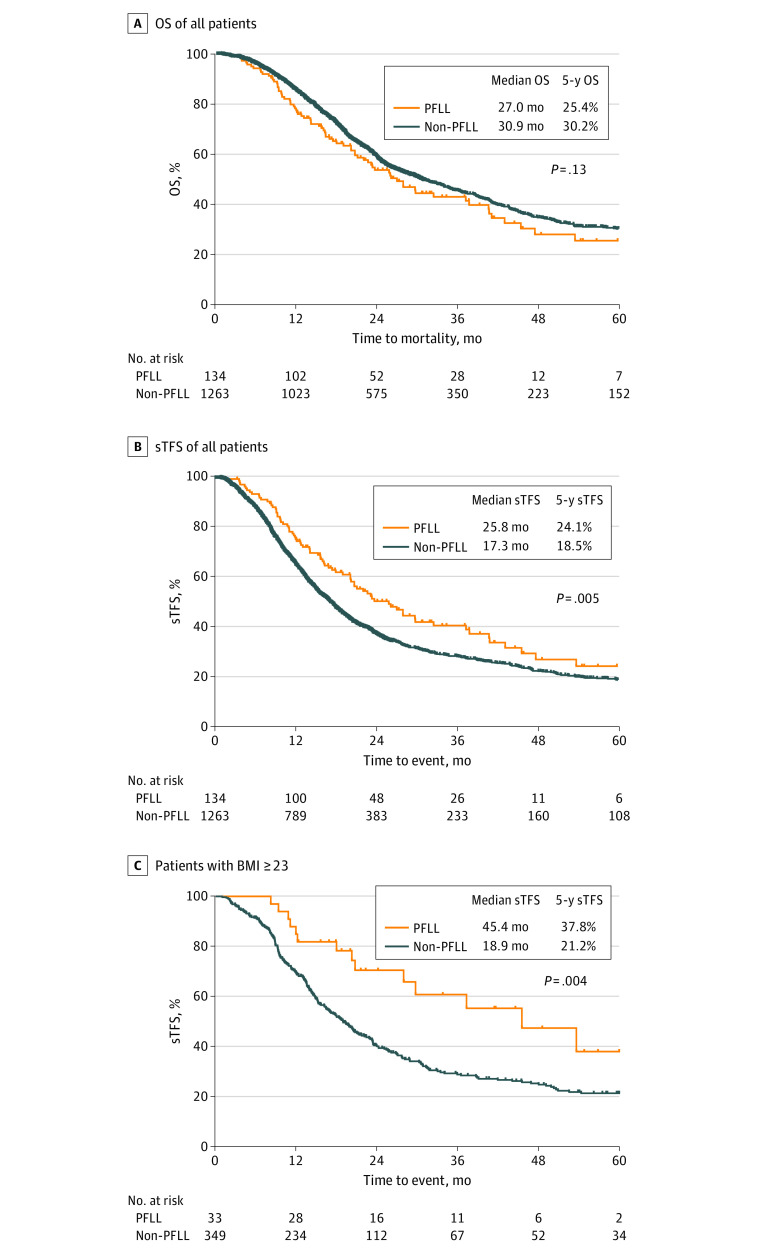
Comparison of Survival Among Patients With Metastatic Nasopharyngeal Carcinoma Who Did vs Did Not Receive Platinum Plus Low-Dose, Long-term Fluorouracil (PFLL) BMI indicates body mass index (calculated as weight in kilograms divided by height in meters squared); OS, overall survival; sTFS, subsequent-line, treatment-free survival.

The median OS was 30.4 (95% CI, 27.4-33.4) months. The OS rate at 1 year was 84.7% (95% CI, 82.8%-86.6%), at 3 years it was 45.1% (95% CI, 42.3%-48.2%), and at 5 years it was 29.7% (95% CI, 26.8%-33.0%). The survival curves for the various regimens are shown in Figure 1 and in eFigure 2 in the Supplement. The OS of patients in the PFLL group was similar to the non-PFLL group (Figure 1A), consistent with the results of multivariable Cox models (eg, mortality using model 2: HR, 0.85; 95% CI, 0.65-1.10; *P* = .22) (Table 2). More specifically, the median OS of patients receiving PFLL (27.0 [95% CI, 22.0-40.6] months) was similar to that of GP (25.7 [95% CI, 22.9-32.9] months), PFSS (26.8 [95% CI, 23.3-31.8] months), and TP (29.8 [95% CI, 25.6-38.8] months), but lower than that of TPF (40.7 [95% CI, 34.7-49.8] months) (eFigure 2B in the Supplement). The 5-year OS rate among patients who received PFLL was 25.4% (95% CI, 16.7%-38.8%), which was not significantly different from that among patients who did not receive PFLL (30.2%; 95% CI, 27.1%-33.5%; *P* = .13) or who received GP (25.1%; 95% CI, 18.1%-35.0%; *P* = .81), PFSS (23.6%; 95% CI, 18.5%-30.0%; *P* = .80) or TP (28.1%; 95% CI, 22.8%-34.7%; *P* = .99), but was lower than that for patients who received TPF (40.4%; 95% CI, 34.7%-47.1%; *P* = .001). The median sTFS of patients in the PFLL group (25.8 [95% CI, 20.2-37.2] months) was significantly (*P* < .001) longer than that of patients in the non-PFLL group as a whole (17.3 [95% CI, 16.2-18.4] months) or for any other regimen (GP, 15.3 [95% CI, 14.2-19.2] months; PFSS, 15.5 [95% CI, 13.7-17.2] months; and TP, 15.7 [95% CI, 13.9-18.4] months) except TPF (23.4 [95% CI, 19.8-25.8] months) (Figure 1B; eFigure 2D in the Supplement). The 5-year sTFS among patients who received PFLL (24.1%; 95% CI, 15.4%-37.6%) was significantly higher than that among patients who did not receive PFLL (18.5%; 95% CI, 16.1%-21.3%; *P* = .005) or who received GP (14.3%; 95% CI, 9.1%-22.5%; *P* = .001), and similar to that for patients who received TPF (28.0%; 95% CI, 23.0%-34.0%; *P* = .74). Cox proportional hazards models indicated that PFLL was associated with significantly better sTFS than non-PFLL (eg, multivariable model 2: HR, 1.61; 95% CI, 1.24-2.07; *P* < .001) (Table 2). On the basis of the results of our previous work assessing the association between BMI and therapeutic outcome,^25,26^ we analyzed survival according to baseline BMI and found that for patients with a baseline BMI of 23 or greater, the median sTFS for patients in the PFLL group was 45.4 (95% CI, 29.7-66.5) months, whereas the median sTFS was 18.9 (95% CI, 16.4-21.6) months for patients in the non-PFLL group, 16.8 (95% CI, 14.1-25.0) months for patients who received GP, and 21.3 (95% CI, 17.7-27.6) months for patients who received TPF (Figure 1C; eFigure 2D, eTable 3 in the Supplement).

**Table 2.** Univariable and Multivariable Cox Proportional Hazards Regression for Various Outcomes^a^

Outcome and treatment group	Rate	Univariable		Multivariable			
				Model 1^b^		Model 2^c^	
		HR (95% CI)	*P* value	HR (95% CI)	*P* value	HR (95% CI)	*P* value
Mortality per 100 person-years							
PFLL	28.4	1 [Reference]		1 [Reference]		1 [Reference]	
Non-PFLL	23.9	0.83 (0.66-1.06)	.14	0.83 (0.62-1.13)	.24	0.85 (0.65-1.10)	.22
GP	27.0	0.96 (0.71-1.29)	.78	0.82 (0.58-1.18)	.29	0.86 (0.62-1.17)	.33
PFSS	27.8	0.97 (0.74-1.26)	.82	1.00 (0.72-1.39)	.99	0.99 (0.74-1.32)	.94
TP	25.2	0.88 (0.67-1.14)	.33	0.87 (0.62-1.21)	.40	0.89 (0.67-1.18)	.41
TPF	18.4	0.63 (0.48-0.83)	.001	0.68 (0.48-0.95)	.02	0.68 (0.50-0.92)	.01
Subsequent-line, treatment-free survival per 100 person-years							
PFLL	22.4	1 [Reference]		1 [Reference]		1 [Reference]	
Non-PFLL	16.1	1.39 (1.10-1.76)	.005	1.55 (1.16-2.08)	.003	1.61 (1.24-2.07)	<.001
GP	19.1	1.59 (1.20-2.09)	.001	1.46 (1.04-2.04)	.03	1.52 (1.13-2.04)	.006
PFSS	11.9	1.65 (1.28-2.13)	<.001	1.99 (1.45-2.74)	<.001	2.01 (1.52-2.66)	<.001
TP	13.2	1.48 (1.15-1.91)	.002	1.58 (1.15-2.17)	.005	1.71 (1.30-2.25)	<.001
TPF	16.5	1.05 (0.81-1.35)	.74	1.29 (0.94-1.78)	.12	1.29 (0.97-1.71)	.08
Radiotherapy per 100 person-months^d^							
PFLL	59.6	1 [Reference]		1 [Reference]		1 [Reference]	
Non-PFLL	2.6	0.08 (0.06-0.09)	<.001	0.06 (0.04-0.08)	<.001	0.07 (0.06-0.10)	<.001
GP	1.2	0.03 (0.02-0.05)	<.001	0.03 (0.02-0.05)	<.001	0.05 (0.03-0.07)	<.001
PFSS	3.1	0.08 (0.06-0.11)	<.001	0.07 (0.05-0.09)	<.001	0.08 (0.06-0.11)	<.001
TP	2.4	0.07 (0.06-0.10)	<.001	0.06 (0.04-0.08)	<.001	0.08 (0.06-0.10)	<.001
TPF	3.1	0.10 (0.08-0.13)	<.001	0.07 (0.05-0.09)	<.001	0.09 (0.06-0.11)	<.001

Abbreviations: BMI, body mass index (calculated as weight in kilograms divided by height in meters squared); GP, cisplatin plus gemcitabine; HR, hazard ratio; PFLL, platinum plus continuous intravenous infusion of low-dose, long-term fluorouracil; PFSS, cisplatin plus fluorouracil with a short term and standard dose; TP, cisplatin plus taxane; TPF, cisplatin plus taxane plus fluorouracil.

^a^ Model 1 and model 2 were adjusting for chemotherapy (non-PFLL vs PFLL) or for chemotherapy (GP vs PFLL, PFSS vs PFLL, TP vs PFLL, TPF vs PFLL), age (≥46 years vs <46 years), sex (female vs male), year of metastasis (2006-2011 vs 2012-2017), pathology findings (nonkeratinizing vs keratinized), number of treatment failures before metastasis (1 vs 0; ≥2 vs 0), T stage (T1 vs T0; T2 vs T0; T3 vs T0; T4 vs T0), N stage (N1 vs N0; N2 vs N0; N3 vs N0), bone metastasis (yes vs no), liver metastasis (yes vs no), lung metastasis (yes vs no), distant lymph node metastasis (single region vs no, multiple regions vs no), number of metastatic sites (multiple vs solitary), and BMI (≥23 vs <23).

^b^ Model 1 excluded patients with missing baseline BMI (n = 230).

^c^ Model 2 included patients with multiple imputation for baseline BMI (n = 230).

^d^ Patients with missing data for radiotherapy during the period of first-line treatment were excluded (n = 94), and the time interval was calculated from treatment initiation to radiotherapy initiation.

**Figure 2.**
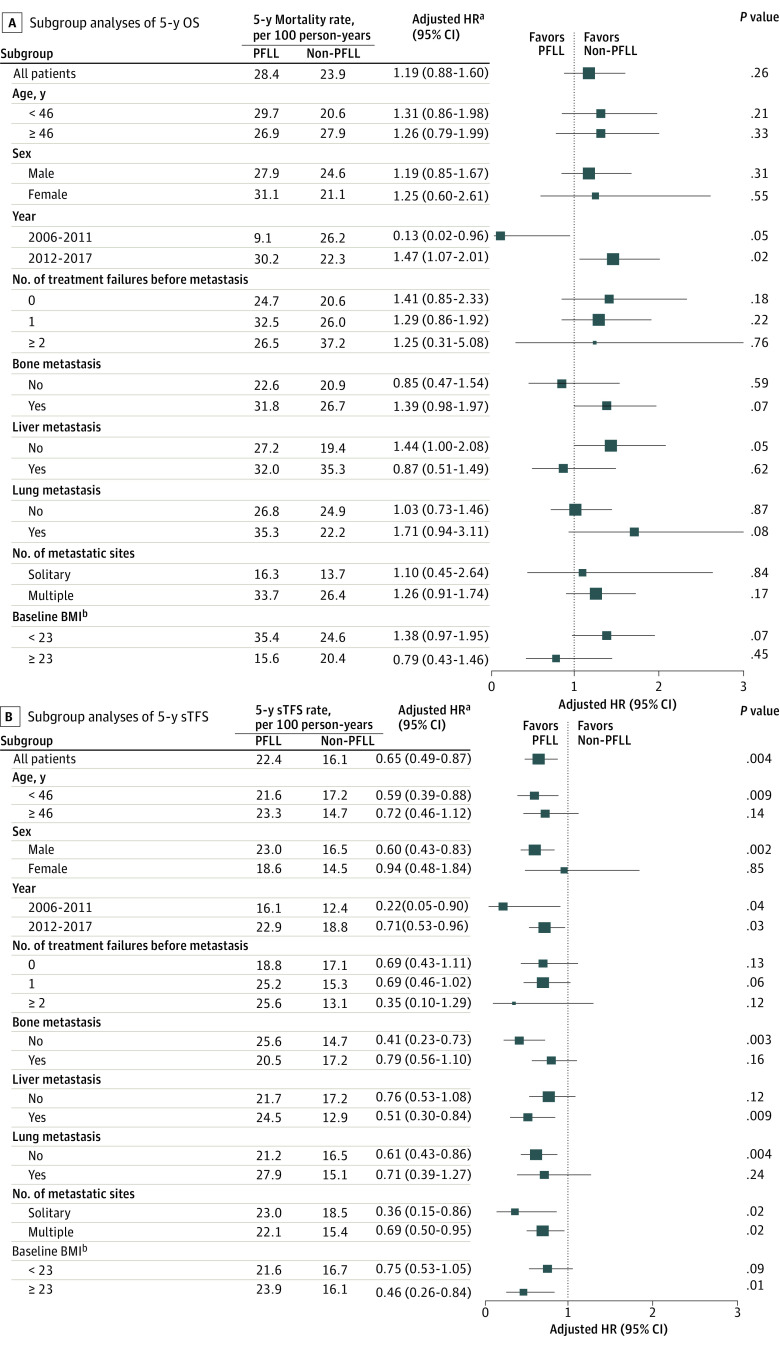
Forest Plots of Subgroup Analyses With Adjusted Hazard Ratios (HRs) Among Patients Who Did vs Did Not Receive Platinum Plus Low-Dose, Long-term Fluorouracil (PFLL) BMI indicates body mass index (calculated as weight in kilograms divided by height in meters squared); OS, overall survival; sTFS, subsequent-line, treatment-free survival. ^a^Multivariable Cox proportional hazard regression models were used to estimate HRs and 95% CIs of OS or sTFS, adjusting for chemotherapy (PFLL vs non-PFLL), age (≥46 years vs <46 years), sex (female vs male), year of metastasis (2006-2011 vs 2012-2017), pathology results (nonkeratinizing vs keratinized), number of treatment failures before metastasis (1 vs 0; ≥2 vs 0), T stage (T1-T4 vs T0), N stage (N1-N3 vs N0), bone metastasis (yes vs no), liver metastasis (yes vs no), lung metastasis (yes vs no), distant lymph node metastasis (single region vs no; multiple regions vs no), number of metastatic sites (multiple vs solitary), baseline BMI (≥23 vs <23). ^b^Patients with missing baseline BMI data were excluded.

To gain further understanding of the potential factors that might be associated with therapeutic outcomes for the PFLL and non-PFLL regimens, we performed subgroup analyses with multivariate Cox proportional hazards models using various clinical parameters (Figure 2). Of note, compared with patients in the non-PFLL group, patients in the PFLL group possibly exhibited better OS at the early time period (2006-2011), with a 5-year mortality rate of 9.1 per 100 person-years vs 26.2 per 100 person-years (adjusted HR, 0.13; 95% CI, 0.02-0.96; *P* = .05) (Figure 2A; eFigure 3 in the Supplement); improved sTFS for patients in the PFLL group was found for most subgroups (Figure 2B).

### Hematologic Toxic Effects and Survival to Toxicity Ratio

The cumulative incidence rate of severe myelosuppression of PFLL was 40.3% (54 of 134), lower than that of GP (50.0% [94 of 188]) and TPF (44.7% [153 of 342]) (Table 3; eTable 4 in the Supplement). The median (IQR) interval from treatment initiation to severe hematologic toxic effects of PFLL was 3.7 (1.5-7.3) months, significantly longer than that of GP (HR, 1.51 [95% CI, 1.09-2.12]; *P* = .02). To quantitatively evaluate survival vs adverse toxic effects, we calculated the STR, with person-year rate of OS and person-year rate of severe hematologic toxic effects. The STR values were 0.81 for PFLL, 0.54 for non-PFLL, 0.41 for GP, 0.57 for PFSS, 0.61 for TP, and 0.65 for TPF.

**Table 3.** Hematologic Toxic Effects and STR

Treatment group	Overall survival		Severe hematologic toxic effects				STR
	No./total No. (%)	Person-year rate, %	No./total No. (%)	Person-year rate, %	Time to severe toxic effects, median (IQR), mo	HR (95% CI)	
PFLL	59/134 (44.0)	22.4	54/134 (40.3)	27.8	3.7 (1.5-7.3)	[Reference]	0.81
Non-PFLL	574/1263 (45.4)	19.9	483/1167 (41.4)	36.7	1.5 (0.7-2.8)	1.19 (0.90-1.58)	0.54
GP	96/203 (47.3)	24.2	94/188 (50.0)	59.8	1.7 (0.5-2.7)	1.51 (1.09-2.12)	0.41
PFSS	126/330 (38.2)	17.2	113/309 (36.6)	30.1	1.5 (0.7-2.8)	1.02 (0.74-1.41)	0.57
TP	159/366 (43.4)	19.4	123/328 (37.5)	31.8	1.5 (0.4-3.1)	1.07 (0.78-1.47)	0.61
TPF	193/364 (53.0)	20.7	153/342 (44.7)	32.0	1.7 (0.5-2.9)	1.30 (0.96-1.78)	0.65

Abbreviations: GP, cisplatin plus gemcitabine; HR, hazard ratio; PFLL, platinum plus continuous intravenous infusion of low-dose, long-term fluorouracil; PFSS, cisplatin plus fluorouracil with a short term and standard dose; STR, survival to toxicity ratio; TP, cisplatin plus taxane; TPF, cisplatin plus taxane plus fluorouracil.

### Radiotherapy

Because the use of PFLL was associated with greater sTFS than standard treatment regimens and less toxicity than GP, radiotherapy could be performed earlier and safely. Thus, we compared the rates of PFLL and non-PFLL regimens in combination with radiotherapy use and found that 133 of 134 patients (99.3%) receiving PFLL underwent radiotherapy. This rate was much higher than that of the non-PFLL groups (non-PFLL, 368 of 1181 [31.2%]; GP, 31 of 187 [16.6%]; PFSS, 99 of 314 [31.5%]; TP, 98 of 340 [28.8%]; and TPF, 140 of 340 [41.2%]). The Cox proportional hazards models showed that receipt of PFLL was significantly associated with receipt of radiotherapy (eg, multivariable model 2: HR for non-PFLL vs PFLL, 0.07; 95% CI, 0.06-0.10; *P* < .001) (Table 2).

## Discussion

Significant progress has been made in the treatment of mNPC during the last decades (eTable 5 in the Supplement), but room for further improving survival of patients with mNPC still exists. In addition to survival outcomes, toxic effects and quality of life should also be considered to obtain a favorable balance between these clinical parameters.^27^ To this end, we evaluated the survival outcomes and toxic effects associated with the use of various common platinum-based chemotherapy regimens in 1397 consecutive patients with mNPC.

Our cohort study found that the median OS of the whole cohort was 30.4 months, and TPF had the best OS (40.7 months) compared with other regimens. However, the triple drug combination TPF failed to improve prognosis in other studies with a higher incidence of intolerable adverse events.^13,28^ Thus, TPF appears to not be the best treatment option for mNPC in clinical practice owing to the high risk of substantial toxic effects. In our study, when sTFS was used as a measure to evaluate the need for subsequent-line therapy after the initial treatment, there was no survival advantage observed in the TPF group, whereas the prolonged median sTFS of PFLL appeared promising. Thus, the survival benefit of TPF was likely confounded by subsequent therapy, whereas the OS in the PFLL group was mainly associated with first-line therapy (Figure 1A and B).

Continuous infusion of fluorouracil was first used in the 1960s. Early studies found that fluorouracil could be infused for up to 60 days without interruption at a dosage of 300 mg/m^2^/d or less.^22,29^ A low-dose venous infusion of fluorouracil for more than 30 days was shown to improve the survival of patients with rectal cancer, with acceptable toxicity, along with concomitant radiotherapy.^30,31^ In our study, administration of fluorouracil at a dosage of 200 mg/m^2^/d for 30 days plus platinum was associated with a clear therapeutic benefit, with prolonged sTFS and tolerable toxicity. Such survival benefits were observed especially in patients with higher baseline BMI during treatment. Among all regimens evaluated in patients with BMIs of 23 or greater, survival in the PFLL treatment group was longest, with a median sTFS of 45.4 months or a 5-year OS rate of 43.1%, which may have been associated with the low toxicity and thus better tolerance of continuous chemotherapy.^32^ Therefore, in clinical practice, supportive care and proper nutrition to enhance patient tolerability to chemotherapy may be a plausible approach to improve the outcome of patients with mNPC. Subgroup analyses showed that the mortality of patients receiving PFLL increased significantly at the later time period (2012-2017), which may be associated with the multidisciplinary synthetic therapy, supportive care, subsequent-line therapies, and immunotherapy in the non-PFLL treatment group; however, sTFS within the PFLL group was still better, likely owing to the low toxicity of PFLL.

To quantitatively evaluate the association between therapeutic outcome and toxicity among the various regimens, we used the STR value as an indicator that reflected both the gain in OS and the cost of severe toxic effects. This quantitative value enabled a practical comparison between the different regimens. For example, the STR for PFLL was the highest among all the regimens evaluated, indicating that PFLL may have a favorable benefit to risk ratio. Although the person-year rates of OS for PFLL and non-PFLL regimens were comparable, severe toxic effects were delayed, as the time interval without severe hematologic adverse events was longer. We speculated that lower toxicity may reduce the burden of treatment-associated costs and potentially improve quality of life. Therapeutic regimens with low toxicity are preferable for patients whose health conditions are too poor to sustain intensive chemotherapy. Moreover, compared with other chemotherapeutic regimens, PFLL provided a better chance for combination with radiotherapy (higher rates). Recently, a phase 3 study showed that chemotherapy plus radiotherapy significantly improved OS in chemotherapy-sensitive patients with de novo mNPC.^33^ The addition of radiotherapy may enable better control of tumor development and delay the seeding of subsequent tumor clones at distant sites.^33,34^

Many studies suggest that enhancing patient antitumor immunity could be an effective way to prevent and control tumor metastasis.^35,36^ However, chemotherapy is usually considered suppressive to the immune system owing to its cytotoxicity against hematopoietic and immune cells^37^ although some researchers believe that chemotherapy may be an adjuvant for antitumor immunity.^38^ Because low-dose fluorouracil has been shown to have immunomodulatory effects by selectively inhibiting the function of myeloid-derived suppressor cells^39,40^ and cisplatin may stimulate T cell function against cancer cells,^41^ it is possible that the PFLL regimen could have a positive effect on the immune system against NPC cells. Further study is needed to test this possibility.

### Strengths and Limitations

A strength of this study is the large sample size of patients with long-term follow-up. The availability of clinical data from a large number of patients with mNPC within a single institution has the advantage of high data comparability owning to uniformity in data collection and recording. This data uniformity enabled us to compare the survival outcomes and toxicity of different regimens associated with treatment of mNPC. In the absence of randomized clinical trial data, our analysis of large sets of real-world data from patients may have important clinical implications.

This study had several limitations. First, owing to the retrospective nature of the analyses, resultant potential bias from selection and imbalance in confounders may exist. Second, our analyses of data across 12 years may complicate data interpretation because changes in clinical practice during this period may affect therapeutic outcome. Third, we were unable to obtain the number of metastatic sites or Epstein-Barr virus DNA copy number, which has been associated with survival outcomes in mNPC. In addition, data on nonhematologic toxic effects and quality of life were limited and thus insufficient to fully evaluate the safety profiles of various chemotherapeutic regimens.

## Conclusions

This retrospective cohort study showed that administration of PFLL was associated with therapeutic benefit for treatment of mNPC. Compared with standard therapeutic regimens, PFLL had similar OS but with significantly longer sTFS and better STR, possibly indicating less severe hematologic toxic effects. The potential stimulatory effect of low-dose fluorouracil and cisplatin on immune cell function could provide additional benefits, but will require further study. Overall, our findings suggest that PFLL may be an option for use as a first-line treatment for patients with mNPC.

## Notes

Supplement. **eFigure 1.** Flowchart **eFigure 2.** Survival Analyses of Patients in the Whole Cohort and Different Subgroups **eFigure 3.** Overall Survival of Patients in Early and Late Time Periods Under Different Treatment **eTable 1.** Clinical Trials About Metastatic Nasopharyngeal Carcinoma at the Sun Yat-sen University Cancer Center (2006-2017) **eTable 2.** Details of Different Chemotherapy Regimens **eTable 3.** Hazard Ratios for Subsequent-Line Treatment-Free Survival of BMI (≥23 kg/m^2^ vs <23 kg/m^2^) **eTable 4.** Severe Hematological Adverse Events in Detail **eTable 5.** Summary of Studies About Systemic Chemotherapy in Metastatic Nasopharyngeal Carcinoma
eReferences.
