# Supplementary material for: Treatment and Survival Outcomes Associated With Platinum Plus Low-Dose, Long-term Fluorouracil for Metastatic Nasopharyngeal Carcinoma
Source: JAMA Netw Open. 2021 Dec 13;4(12):e2138444. doi: 10.1001/jamanetworkopen.2021.38444 (PMC8669524; doi:10.1001/jamanetworkopen.2021.38444)
Supplement: Supplement. — eFigure 1. Flowchart eFigure 2. Survival Analyses of Patients in the Whole Cohort and Different Subgroups eFigure 3. Overall Survival of Patients in Early and Late Time Periods Under Different Treatment eTable 1. Clinical Trials About Metastatic Nasopharyngeal Carcinoma at the Sun Yat-sen University Cancer Center (2006-2017) eTable 2. Details of Different Chemotherapy Regimens eTable 3. Hazard Ratios for Subsequent-Line Treatment-Free Survival of BMI (≥23 kg/m2 vs <23 kg/m2) eTable 4. Severe Hematological Adverse Events in Detail eTable 5. Summary of Studies About Systemic Chemotherapy in Metastatic Nasopharyngeal Carcinoma eReferences. [file jamanetwopen-e2138444-s001.pdf]

Supplementary Online Content

Zheng SH, Liu SR, Wang HB, et al. Treatment and survival outcomes associated with platinum plus low-dose, long-term fluorouracil for metastatic nasopharyngeal carcinoma. *JAMA Netw Open*. 2021;4(12):e2138444. doi:10.1001/jamanetworkopen.2021.38444

eFigure 1. Flowchart
eFigure 2. Survival Analyses of Patients in the Whole Cohort and Different Subgroups
eFigure 3. Overall Survival of Patients in Early and Late Time Periods Under Different Treatment
eTable 1. Clinical Trials About Metastatic Nasopharyngeal Carcinoma at the Sun Yat-sen University Cancer Center (2006-2017)
eTable 2. Details of Different Chemotherapy Regimens
eTable 3. Hazard Ratios for Subsequent-Line Treatment-Free Survival of BMI (≥23 kg/m^2 vs <23 kg/m^2)
eTable 4. Severe Hematological Adverse Events in Detail
eTable 5. Summary of Studies About Systemic Chemotherapy in Metastatic Nasopharyngeal Carcinoma
eReferences.

This supplementary material has been provided by the authors to give readers additional information about their work.

© 2021 Zheng SH et al. *JAMA Network Open*.

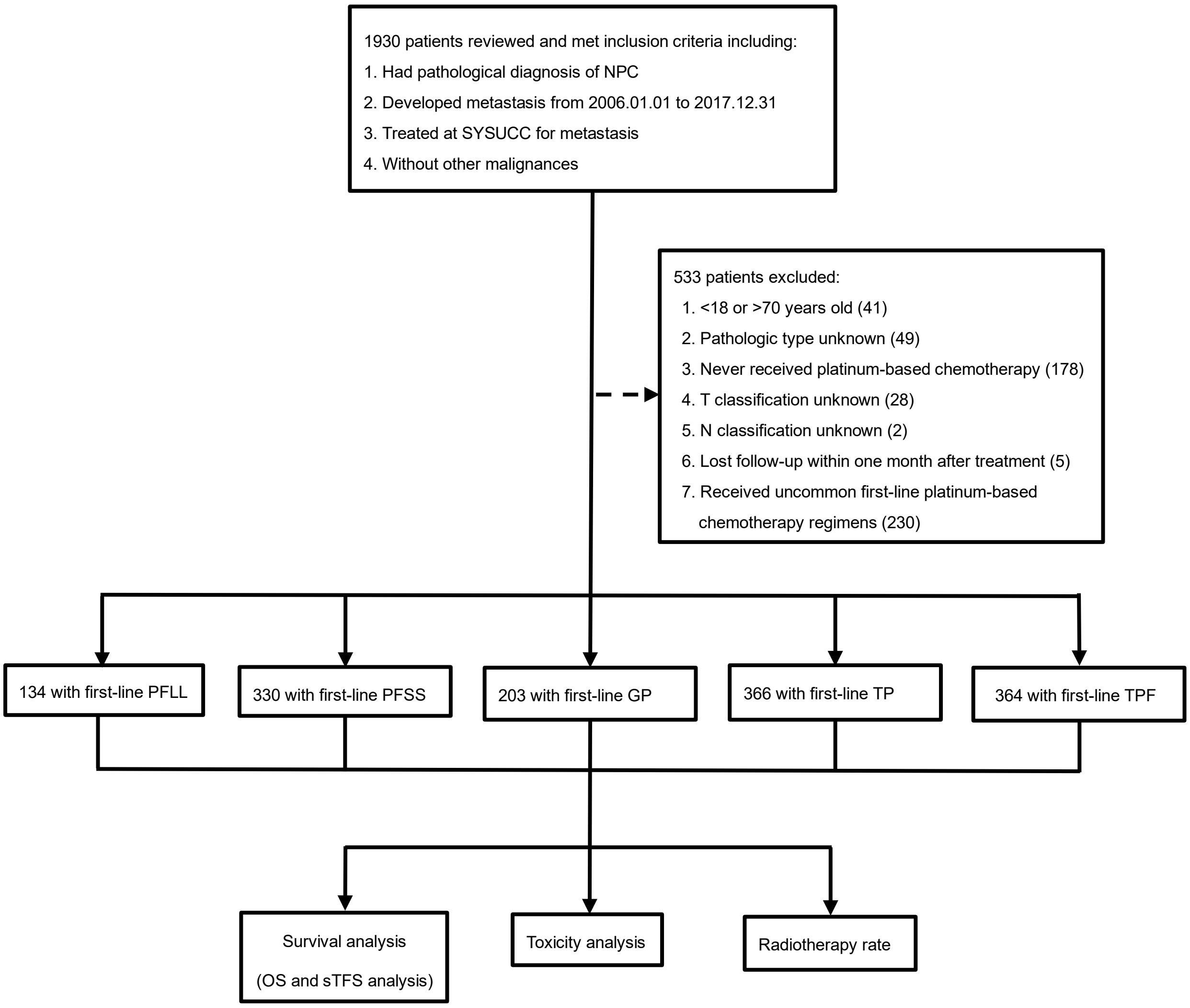

eFigure 1. Flowchart

Abbreviations: GP, cisplatin plus gemcitabine; NPC, nasopharyngeal carcinoma; OS, overall survival; PFLL, platinum plus continuous intravenous infusion of low-dose, long-term fluorouracil; PFSS, cisplatin plus fluorouracil with a short term and standard dose; sTFS, subsequent-line treatment-free survival; SYSUCC, Sun Yet-sen University Cancer Center; TP, cisplatin plus taxane; TPF, cisplatin plus taxane plus fluorouracil.

© 2021 Zheng SH et al. *JAMA Network Open*.

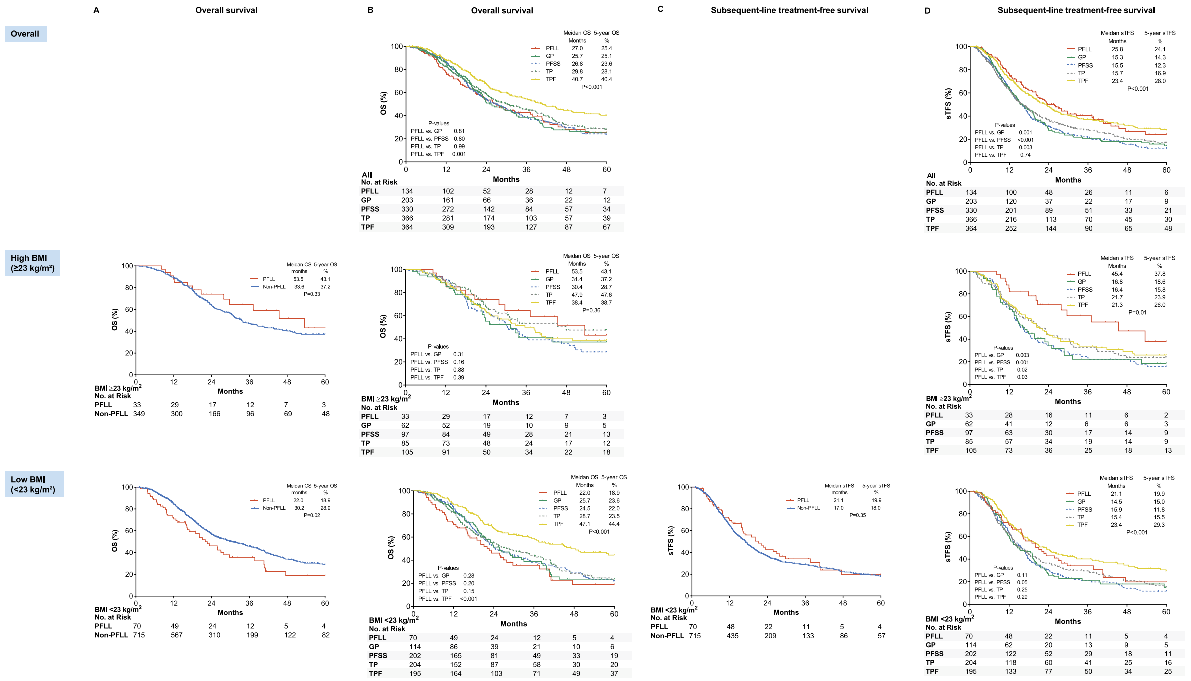

eFigure 2. Survival Analyses of Patients in the Whole Cohort and Different Subgroups

Panel A shows overall survival (OS) of patients under PFLL and non-PFLL in different subgroups. Panel B shows OS of patients with different chemotherapy regimens in the whole cohort and different subgroups. Panel C shows subsequent-line treatment-free survival (sTFS) of patients under PFLL and non-PFLL with low baseline BMI. Panel D shows sTFS of patients with different chemotherapy regimens in the whole cohort and different subgroups.

Subgroup survival analyses included patients with records of high baseline BMI (≥23 kg/m²), low baseline BMI (<23 kg/m²).

Abbreviations: BMI, body mass index (calculated as weight in kilograms divided by height in meters squared); GP, cisplatin plus gemcitabine; PFLL, platinum plus continuous intravenous infusion of low-dose, long-term fluorouracil; PFSS, cisplatin plus fluorouracil with a short term and standard dose; TP, cisplatin plus taxane; TPF, cisplatin plus taxane plus fluorouracil.

© 2021 Zheng SH et al. *JAMA Network Open*.

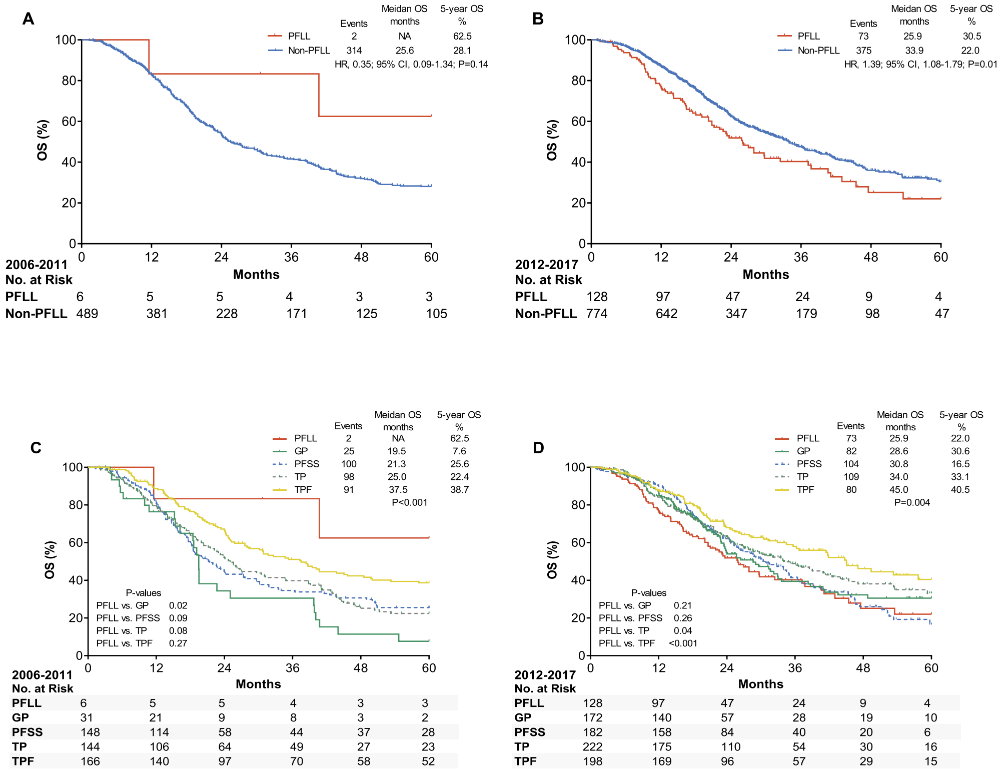

eFigure 3. Overall Survival of Patients in Early and Late Time Periods Under Different Treatment

Panel A and panel C shows overall survival (OS) analysis of patients in the early time period (2006-2011). Panel B and panel D shows OS analysis of patients in the late time period (2012-2017).

Abbreviations: GP, cisplatin plus gemcitabine; PFLL, platinum plus continuous intravenous infusion of low-dose, long-term fluorouracil; PFSS cisplatin plus fluorouracil with a short term and standard dose; TP, cisplatin plus taxane; TPF, cisplatin plus taxane plus fluorouracil.

© 2021 Zheng SH et al. *JAMA Network Open*.

eTable 1. Clinical Trials About Metastatic Nasopharyngeal Carcinoma at the Sun Yat-sen University Cancer Center (2006-2017)

ClinicalTrails.gov reference	status	year	treatment	No.	publications (doi)
NCT00630149	Completed	2007-2012	Pemetrexed	35	
NCT00747799	Completed	2009-2011	Sorafenib+P+F	54	
NCT01462903	Unknown	2011-2014	T cell immunoterhapy	<20	
NCT01392235	Completed	2011-2016	Famitinib	58	
NCT01655628	Unknown	2012-2014	G+CBP+CIK cells<br>G+CBP	40	
NCT01735409	Unknown	2012-2014	ABX+P (1-day, 2-day, or 3-day regimen)	69	10.1186/s12885-016-2517-5.
NCT01616849	Completed	2012-2015	P+F+nimotuzumab	39	10.1093/annonc/mdz020
NCT01528618	Active, not recruiting	2012-2017	G+P<br>P+F	362	10.1016/S0140-6736(16)31388-5
NCT02111460	Active, not recruiting	2013-2022	Chemotherapy +Radiotherapy<br>Chemotherapy Alone	126	10.1001/jamaoncol.2020.1808
NCT02115958	Completed	2014-2015	Non-cancer stem cell vaccine<br>Cancer stem cell vaccine (low, middle, high dose)	40	
NCT02250599	Unknown	2014-2016	CBP+T<br>AVASTIN+CBP+T	80	
NCT02590133	Recruiting	2015-2020	NDP+F+Endostar<br>NDP+F	328	
NCT02460419	Recruiting	2015-2021	Maintenance CAP +best supportive care<br>Best supportive care	176	
NCT02633176	Recruiting	2015-2023	P+D<br>Cetuximab+P+D	120	10.3389/fonc.2020.01011.
NCT03007836	Completed	2016-2019	High-activity natural killer	20	
NCT02605967	Active, not recruiting	2016-2020	PDR001<br>Chemotherapy	122	

© 2021 Zheng SH et al. *JAMA Network Open*.

eTable 1 (continued). Clinical trials about metastatic nasopharyngeal carcinoma at the Sun Yat-sen University Cancer Center (2006-2017).

ClinicalTrails.gov reference	status	year	treatment	No.	publications (doi)
NCT02915432	Active, not recruiting	2016-2021	JS001 (different dose)	<403	
NCT04476641	Recruiting	2016-2022	DC-CIK	<686	
NCT03260179	Unknown	2017-2019	AL3810	<60	
NCT03210389	Not yet recruiting	2017-2021	Lobaplatin+F	60	

Abbreviations: ABX, Abraxane; CAP, capecitabine; CBP, carboplatin; CIK, cytokines induced killer; D, docetaxel; DC, dendritic cell; F, fluorouracil; G, gemcitabine; NDP, nedaplatin; P, cisplatin; T, paclitaxel.

© 2021 Zheng SH et al. *JAMA Network Open*.

eTable 2. Details of Different Chemotherapy Regimens

Regimen	Drug	Dose	Day	Route	Cycle Frequency
PFLL	fluorouracil	200 mg/m^2/day	1-30	continuous IV infusion	60 days
	cisplatin or nedaplatin or lobaplatin	70 mg/m^2/ or 80 mg/m^2 or 30 mg/m^2	1, 28	IV infusion	
GP	gemcitabine	1,000 mg/m^2	1, 8	IV infusion	21 days
	cisplatin	80 mg/m^2	1	IV infusion	
PFSS	fluorouracil	1,000 mg/m^2/day	1-4	continuous IV infusion	21 days
	cisplatin	80 mg/m^2	1	IV infusion	
TP	paclitaxel or docetaxel	175 mg/m^2 or 75 mg/m^2 or	1	IV infusion	21 days
	cisplatin	75 mg/m^2	1	IV infusion	
TPF	paclitaxel or docetaxel	135~175 mg/m^2 or 60~75 mg/m^2	1	IV infusion	21 days
	cisplatin	60~75 mg/m^2	1	IV infusion	
	fluorouracil	600~750 mg/m^2/day	1-5	continuous IV infusion	

Abbreviations: GP, cisplatin plus gemcitabine; IV, intravenous; PFLL, platinum plus continuous intravenous infusion of low-dose, long-term fluorouracil; PFSS, cisplatin plus fluorouracil with a short term and standard dose; TP, cisplatin plus taxane; TPF, cisplatin plus taxane plus fluorouracil.

© 2021 Zheng SH et al. *JAMA Network Open*.

eTable 3. Hazard Ratios for Subsequent-Line Treatment-Free Survival of BMI (≥23 kg/m^2 vs <23 kg/m^2)

Chemotherapy	Hazard ratio	95% confidence interval	P-values
PFLL	0.47	0.26-0.86	**0.01**
Non-PFLL	0.82	0.68-0.98	**0.03**
GP	0.88	0.61-1.28	0.50
PFSS	0.87	0.66-1.14	0.30
TP	0.79	0.59-1.07	0.12
TPF	1.10	0.81-1.48	0.54

Abbreviations: GP, cisplatin plus gemcitabine; PFLL, platinum plus continuous intravenous infusion of low-dose, long-term fluorouracil; PFSS, cisplatin plus fluorouracil with a short term and standard dose; TP, cisplatin plus taxane; TPF, cisplatin plus taxane plus fluorouracil.

© 2021 Zheng SH et al. *JAMA Network Open*.

eTable 4. Severe Hematological Adverse Events in Detail

	Leukopenia		Neutropenia		Thrombocytopenia		Any toxic effect[a]	
	No. (%)	**P-values[b]**	***No. (%)***	**P-values[b]**	***No. (%)***	**P-values[b]**	***No. (%)***	**P-values[b]**
PFLL (N=134)	36 (26.9)	Reference	22 (16.4)	Reference	36 (26.9)	Reference	54 (40.3)	Reference
Non-PFLL (N=1263)	311 (26.6)	0.96	399 (34.2)	**<0.001**	117 (10.0)	<0.001	483 (41.4)	0.81
GP (N=188)	64 (34.0)	0.17	73 (38.8)	**<0.001**	44 (23.4)	0.48	94 (50.0)	0.09
PFSS (N=309)	50 (16.2)	0.009	94 (30.4)	**0.002**	19 (6.1)	<0.001	113 (36.6)	0.46
TP (N=328)	92 (28.0)	0.80	102 (31.1)	**0.001**	27 (8.2)	<0.001	123 (37.5)	0.57
TPF (N=342)	105 (30.7)	0.41	130 (38.0)	**<0.001**	27 (7.9)	<0.001	153 (44.7)	0.38

[a] Any toxic effect included grade 3 or higher leukopenia, neutropenia or thrombocytopenia.

[b] P-values were calculated by the log-rank test.

Abbreviations: GP, cisplatin plus gemcitabine; PFLL, platinum plus continuous intravenous infusion of low-dose, long-term fluorouracil; PFSS, cisplatin plus fluorouracil with a short term and standard dose; TP, cisplatin plus taxane; TPF, cisplatin plus taxane plus fluorouracil.

© 2021 Zheng SH et al. *JAMA Network Open*.

eTable 5. Summary of Studies About Systemic Chemotherapy in Metastatic Nasopharyngeal Carcinoma

Author (Year)	Study	Treatment	No.	mOS (mo)	mPFS (mo)	ORR (%)	≥ grade 3 adverse effect (%)			
							L	N	T	A
Zhang et al (2016)[1]	-China<br>-Multicenter<br>-Phase III RCT	G+P<br>F+P	181<br>181	29.1<br>20.9	7·0<br>5.6	64<br>42	29<br>9	23<br>13	13<br>2	-
Peng et al (2015)[2]	-China<br>-Multicenter<br>-Phase II	D+NDP	78	15.7	7.9	65.8	-	15.4	0	7.7
Jin et al (2014)[3]	-China<br>- Single center<br>-Phase II	G+P+Endo	30	1-year, 90.2%	19.4	85.7	-	46.4	0	14.3
Xue et al (2013)[4]	-China<br>-Phase II	P+F+Sorafinib	54	11.8	7.2	77.8	85.2	63	64.9	74.1
Chen et al (2013)[5]	-China<br>-Single center<br>-Phase II	T+P+F	95	22.7	8.6	78.9	14.7	17.9	6.4	3.2
You et al (2012)[6]	-Canada<br>-Phase II	G+P/CBP+ Erotinib	20	1-year, 80%	6.3	37	-	63	37	21
Chua et al (2012)[7]	-Asia<br>-Multicenter<br>-Phase II	P+CAP	39	28.0	7.3	53.8	-	50	9	5
Ji et al (2012)[8]	-Korea<br>-Multicenter<br>-Phase II	D+P	47	28.5	9.6	70.2	-	0.8	-	0.8
Ma et al (2009)[9]	-Hong Kong<br>-Multicenter<br>-Phase II	G+OXP	40	19.6	9	56.1	22	22	17	2
Leong et al (2008)[10]	-Singapore<br>-Retro	G+T+CBP ±(F+LV)	28	22	8	86	-	79	29	32
Li et al (2008)[11]	-China<br>-Multicenter<br>-Phase II	P+CAP	48	13.3	7.7	63	-	14.6	2.1	4.2
Chua et al (2005)[12]	-Hong Kong<br>-Phase II	D+P	19	12.4	5.6	62.5	79.5	100	11	20-30
Leong et al (2005)[13]	-Singapore<br>-Phase II	T+CAP+G	32	18.6	8.1	78	-	78	41	41
Ciuleanu et al (2004)[14]	-Greece<br>-Multicenter<br>-Phase II	T+CBP	40	11.5	3.5	27.5	17.5	7.5	10	17.5
McCarthy et al (2002)[15]	-Canada<br>-Phase II<br>-Closed early	D+P	9	1-year, 76%	8.4	22	-	100	0	11

© 2021 Zheng SH et al. *JAMA Network Open*.

Abbreviations: A, anemia; CAP, capecitabine; CBP, carboplatin; D, docetaxel; Endo, Endostar; F, fluorouracil; G, gemcitabine; L, leukopenia; LV, leucovorin; mo, months; mOS, median overall survival; mPFS, median progression-free survival; N, neutropenia; NDP, nedaplatin; ORR, overall response rate; OXP, oxaliplatin; P, cisplatin; RCT, randomized clinical trial; Retro, retrospective study; T, thrombocytopenia; T, paclitaxel.

© 2021 Zheng SH et al. *JAMA Network Open*.

eReferences

1. Zhang L, Huang Y, Hong S, et al. Gemcitabine plus cisplatin versus fluorouracil plus cisplatin in recurrent or metastatic nasopharyngeal carcinoma: a multicentre, randomised, open-label, phase 3 trial. Lancet 2016;388:1883-92.
2. Peng PJ, Lv BJ, Tang C, et al. Phase II trial of docetaxel combined with nedaplatin for patients with recurrent and metastatic nasopharyngeal carcinoma. Drug Des Devel Ther 2015;9:6401-5.
3. Jin T, Li B, Chen XZ. A phase II trial of Endostar combined with gemcitabine and cisplatin chemotherapy in patients with metastatic nasopharyngeal carcinoma (NCT01612286). Oncol Res 2013;21:317-23.
4. Xue C, Huang Y, Huang PY, et al. Phase II study of sorafenib in combination with cisplatin and 5-fluorouracil to treat recurrent or metastatic nasopharyngeal carcinoma†. Ann Oncol 2013;24:1055-61.
5. Chen C, Wang F, An X, et al. Triplet combination with paclitaxel, cisplatin and 5-FU is effective in metastatic and/or recurrent nasopharyngeal carcinoma. Cancer Chemoth Pharm 2013;71:371-8.
6. You B, Le Tourneau C, Chen EX, et al. A phase II trial of Erlotinib as maintenance treatment after gemcitabine plus platinum-based chemotherapy in patients with recurrent and/or metastatic nasopharyngeal carcinoma. Am J Clin Oncol 2012;35:255-60.
7. Chua DT, Yiu HH, Seetalarom K, et al. Phase II trial of capecitabine plus cisplatin as first-line therapy in patients with metastatic nasopharyngeal cancer. Head Neck 2012;34:1225-30.
8. Ji JH, Yun T, Kim S, et al. A prospective multicentre phase II study of cisplatin and weekly docetaxel as first-line treatment for recurrent or metastatic nasopharyngeal cancer (KCSG HN07-01). Eur J Cancer 2012;48:3198-204.
9. Ma BBY, Hui EP, Wong SCC, et al. Multicenter phase II study of gemcitabine and oxaliplatin in advanced nasopharyngeal carcinoma--correlation with excision repair cross-complementing-1 polymorphisms. Ann Oncol 2009;20:1854-9.
10. Leong SS, Wee J, Rajan S, et al. Triplet combination of gemcitabine, paclitaxel, and carboplatin followed by maintenance 5-fluorouracil and folinic acid in patients with metastatic nasopharyngeal carcinoma. Cancer-Am Cancer Soc 2008;113:1332-7.
11. Li Y, Wang F, Jiang W, et al. Phase II study of capecitabine and cisplatin combination as first-line chemotherapy in Chinese patients with metastatic nasopharyngeal carcinoma. Cancer Chemoth Pharm 2008;62:539-44.
12. Chua DTT, Sham JST, Au GKH. A phase II study of docetaxel and cisplatin as first-line chemotherapy in patients with metastatic nasopharyngeal carcinoma. Oral Oncol 2005;41:589-95.
13. Leong S, Wee J, Tay MH, et al. Paclitaxel, carboplatin, and gemcitabine in metastatic nasopharyngeal carcinoma. Cancer-Am Cancer Soc 2005;103:569-75.
14. Ciuleanu TE, Fountzilas G, Ciuleanu E, Plataniotis M, Todor N, Ghilezan N. Paclitaxel and carboplatin in relapsed or metastatic nasopharyngeal carcinoma: a multicenter phase II study. J Buon 2004;9:161-5.
15. McCarthy JS, Tannock IF, Degendorfer P, Panzarella T, Furlan M, Siu LL. A Phase II trial of docetaxel and cisplatin in patients with recurrent or metastatic nasopharyngeal carcinoma. Oral Oncol 2002;38:686-90.

© 2021 Zheng SH et al. *JAMA Network Open*.
